# Whole-Genome Sequence of the Novel *Enterobacter* Bacteriophage Arya with an Integrase Pseudogene, Isolated from the Gut of the Formosan Subterranean Termite

**DOI:** 10.1128/genomeA.00838-17

**Published:** 2018-01-04

**Authors:** Chinmay Vijay Tikhe, Chris R. Gissendanner, Claudia Husseneder

**Affiliations:** a Department of Entomology, Louisiana State University Agricultural Center, Baton Rouge, Louisiana, USA; b School of Science, University of Louisiana Monroe, Monroe, Louisiana, USA

## Abstract

We isolated and sequenced the novel *Enterobacter* bacteriophage Arya from termite gut. The genome showed synteny to lytic bacteriophage genomes; however, the genome encodes a truncated, putatively nonfunctional integrase pseudogene. Lysogeny-related genes were previously observed in certain lytic phages, but their role and evolution remain unclear.

## GENOME ANNOUNCEMENT

Previous studies suggest that the termite gut contains a population of unexplored novel bacteriophages (1, 2). In this study, we isolated a novel bacteriophage, Arya, infecting *Enterobacter* sp. CT7 (GenBank accession no. KT204538), which is the third bacteriophage isolated from the termite gut and the second to infect *Enterobacter* sp. CT7.

Bacteriophage Arya was isolated from the gut of the Formosan subterranean termite, *Coptotermes formosanus*. Phage isolation, DNA extraction, DNA sequencing and annotation, and electron microscopy were carried out as described previously (1). Bacteriophage Arya produced small but clear plaques on *Enterobacter* sp. CT7. DNA sequencing produced a linear contig with terminal redundancy. PCR and restriction digestion confirmed the DNA to be circularly permuted. Arya has a genome length of 41,918 bp, with a G+C content of 54%. The genome has a total of 64 predicted protein-coding sequences and an arginine tRNA gene. Of the 64 genes, 55 produced a match in the NCBI GenBank protein nr database. Based on similarity to sequences in the database, 22 proteins were assigned a putative function. The overall genome architecture is conserved, with a packaging module, a structural module, a DNA replication/metabolism module, and a lysis cassette. The genome of phage Arya showed a high level of synteny to *Escherichia* phage vB_EcoM_ECO1230-10 (3), *Escherichia* phage vB_EcoM-ep3 (4), and *Pseudomonas* phage PPpW-3 (5). The *Escherichia* bacteriophages vB_EcoM_ECO1230-10 and vB_EcoM-ep3 are lytic, and no integrase or other lysogeny genes are present in their genomes (3, 4). Despite being lytic, phage PPpW-3 harbors a predicted integrase pseudogene. The Arya genome also contains a predicted integrase pseudogene. It encodes a 43-amino-acid product which is small compared to products of other integrases and is most likely nonfunctional (6). Both the PPpW-3 and Arya genomes contain an arginine tRNA gene located next to the integrase pseudogene. Also, similar to phage PPpW-3, Arya did not show the presence of other essential lysogeny genes (repressor and antirepressor genes). The presence of an integrase pseudogene in phage PPpW-3 is hypothesized to be the result of a horizontal gene transfer event (3). Electron microscopy and VIRFAM (7) analysis confirmed phage Arya to be a member of the *Myoviridae* family.

In another study, we isolated bacteriophage Tyrion, which also infects *Enterobacter* sp. CT7 (2). Phage Tyrion is lysogenic and is predicted to alter the host cell receptors to provide superinfection immunity against the host. Isolation and sequencing of bacteriophage Arya provides us with a model system for studying superinfection immunity and the dynamics among a bacterial host, a lytic phage, and a lysogenic phage.

### Accession number(s).

The complete annotated sequence of the *Enterobacter* phage Arya genome can be accessed under the GenBank accession no. KX231828.
